# The SPARE score reliably predicts the conversion from open partial to radical nephrectomy

**DOI:** 10.3325/cmj.2021.62.464

**Published:** 2021-10

**Authors:** Hakan Bahadir Haberal, Burak Citamak, Mesut Altan, Sertac Yazici, Bulent Akdogan, Haluk Ozen, Ali Ergen

**Affiliations:** Department of Urology, Hacettepe University, Ankara, Turkey

## Abstract

**Aim:**

To assess the power of nephrometry scores to predict the intraoperative conversion from partial nephrectomy (PN) to radical nephrectomy (RN).

**Methods:**

We identified all the patients at our institution who were scheduled for PN between April 2012 and December 2017. Patients who underwent robotic or laparoscopic surgery were excluded. A total of 149 patients (94 men) who underwent open surgery and had complete data were included. The power of the R.E.N.A.L., PADUA, SPARE, and DAP scores to predict the conversion to RN, and the threshold values were assessed. In the multivariate analysis, the predictive power of the nephrometry scores was tested by separately including them in different models.

**Results:**

The median age was 57 (48-67) years, while the median follow-up was 15 (7-29.5) months. The overall conversion rate was 10.7%. The optimal cut-off values for the R.E.N.A.L., PADUA, SPARE, and DAP scores were 7.5, 9.5, 5.5 and 7.5, respectively. The SPARE score had the highest area under the curve (AUC = 0.807, *P* < 0.001). In the multivariate analysis, the SPARE score had the highest odds ratio (OR 12.561; confidence interval 3.456-45.534, *P* < 0.001).

**Conclusion:**

A high SPARE score was significantly associated with the conversion to RN in patients who underwent open PN.

Renal cell carcinoma (RCC) constitutes 2%-3% of adult cancers (1). Although many patients with RCC remain asymptomatic until the late disease stages, the majority are diagnosed at a localized stage (1,2), when surgery is a highly effective curative treatment (1). Surgical treatment options include partial and radical nephrectomy (RN) (1). Partial nephrectomy (PN) is the standard treatment method for patients with T1a tumors, while providing similar results in patients with T1b and T2 tumors (3).

As PN preserves the kidney functions, it decreases the risk of chronic kidney disease (CKD) and subsequent cardiovascular disease risk (4,5). However, PN is a complex surgical procedure with high complication rates compared with RN (6). In recent years, PN has been applied for treatment of complex renal tumors, which carries an increased risk of the conversion to RN (7,8).

Various nephrometry scores have been developed to standardize the reporting of renal mass size in patients scheduled for RCC surgery (9-12). To the best of our knowledge, no study so far has compared the four nephrometry scores in terms of conversion to RN prediction. In this study, we retrospectively tested the power of nephrometry scores to predict the intraoperative conversion from PN to RN in patients with RCC.

## PATIENTS AND METHODS

**Figure 1:**
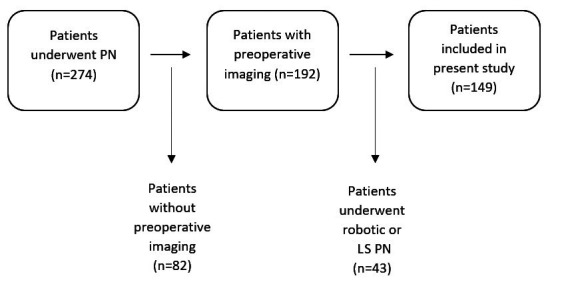
Inclusion process flowchart. LS – laparoscopic; PN – partial nephrectomy.

We retrospectively reviewed the records of 274 consecutive patients who underwent PN due to a solitary renal tumor between April 2012 and December 2017. Patients without preoperative cross-sectional imaging recorded in the hospital information system (n = 82) or those who underwent robotic or laparoscopic surgery (n = 43) were excluded (Figure 1). The data of 149 patients who underwent open PN and had complete data were analyzed. The study was approved by the Ethics Committee of Hacettepe University.

All patients underwent preoperative cross-sectional imaging (abdominal computed tomography [CT] or magnetic resonance imaging [MRI]), and thorax CT to evaluate the presence of lung metastasis at the time of diagnosis. The patients scheduled for PN who subsequently underwent RN were identified. The nephrometry scores were calculated by the same urologist (H.B.H.).

Hemoglobin levels below 12 g/dL for women and below 13 g/dL for men indicated anemia (13). Renal function was assessed with the estimated glomerular filtration rate (eGFR) using the modification of diet in renal disease equation (MDRD) (14). CKD was defined as eGFR<60 mL/min/1.73m^2^. Charlson Comorbidity Index (CCl) was used to determine the patients' general health in the preoperative period (15). Complications that occurred within 30 days after surgery were evaluated according to the Clavien-Dindo complication classification system (16); patients were classified into two groups: minor (Clavien-Dindo 1-2) and major (Clavien-Dindo 3-4) complications group. Blood transfusions were performed intraoperatively due to excessive bleeding or a hemoglobin drop, or postoperatively due to vital signs changes. Tumor complexity was assessed by using the R.E.N.A.L. (maximum tumor diameter, exophytic/endophytic properties, proximity to the collecting system or sinus, and location relative to the polar line), PADUA (longitudinal tumor location, rim location, relationships with the sinus, relationships with the urinary collecting system, percentage of the tumor protruding into the kidney, and maximum tumor diameter), SPARE (rim location, relationships with the sinus, percentage of the tumor protruding into the kidney, and maximum tumor diameter), and DAP (maximum tumor diameter, axial distance, and polar distance) scores (9-12). The R.E.N.A.L. score was considered low if 4–6, intermediate if 7–9, and high if 10–12. The PADUA score was classified as low if 6–7, intermediate if 8–9, and high if ≥10. The SPARE score was classified as low if 0-3, intermediate if 4-7, and high if 8-10. DAP score was classified as low if 3–5 and high if 6–9.

We gathered data on patient demographics and perioperative variables, including age, sex, body mass index, CCI, preoperative CKD, preoperative anemia, hospitalization time, and drain removal time. Tumor size, tumor location, and the R.E.N.A.L., PADUA, SPARE, and DAP scores were obtained by a review of preoperative imaging. Surgical data included estimated blood loss (EBL), intraoperative complications, operative time, and intraoperative transfusion rates. Pathological data included pathological tumor size, histology, nuclear grade, and pathological stage.

PN was performed using an open transperitoneal technique. Depending on the tumor properties, wedge resection or enucleation was performed. Warm hilar ischemia or ultrasound were performed at the primary surgeon's discretion.

The patients were classified into two groups according to the conversion from PN to RN (conversion or no conversion). The two groups were compared in terms of patient demographics and tumor characteristics. The factors affecting the conversion to RN were evaluated in models that included each of the nephrometry scores individually.

### Statistical analysis

The normality of distribution was assessed with the Kolmogorov-Smirnov test. Parametric variables are presented as mean ± standard deviation, while nonparametric variables are presented as median and interquartile range. In the univariate analysis, the χ^2^ test or Fisher exact test were used for the comparison of nominal data, the *t* test for parametric variables, and the Mann-Whitney U test for nonparametric variables. Receiver operating characteristic (ROC) curves were plotted to assess the predictive value of the R.E.N.A.L., PADUA, SPARE, and DAP scores for conversion to RN. The threshold values were determined using the ROC curves, and the value with the highest sensitivity and specificity was calculated. Binary logistic regression analysis and backward stepwise model were used in the multivariate analysis. The significance level was set at *P* < 0.05. The statistical analyses were performed with the SPSS for Windows, version 24.0 (IMB Corp., Armonk, NY, USA).

## RESULTS

The median age was 57 (range 48-67) years. The median follow-up was 15 (range 7-29.5) months. Patients' demographic and preoperative characteristics are shown in Table 1. Of 149 patients who underwent PN, 16 (10.7%) experienced the conversion to RN. The reasons for the conversion were tumor size discordance (the tumor was larger than expected) or suspicion of advanced disease (n = 6, 37.5%), invasion of hilar structures (n = 5, 31.25%), insufficient renal remnant after resection (n = 1, 6.25%), compromised renal arterial supply after resection (n = 1, 6.25%), failure to progress in surgery (n = 1, 6.25%), and failure to achieve clear margins (n = 1, 6.25%). One patient was hospitalized for retroperitoneal bleeding on postoperative day 12, and completion nephrectomy was performed.

**Table 1:** Clinical and preoperative characteristics*

**Parameters**	Entire cohort	Conversion	No conversion	
N (%)	149 (100)	16 (10.7)	133 (89.3)	-
Age in years; median (IQR)	57 (48-67)	59 (51.25-67)	57 (47.5-66.5)	0.602^†^
BMI (kg/m^2^); median (IQR)	27.06 (24.34-31.22)	25.31 (23.33-28)	27.17 (24.35-32.48)	0.148^†^
Charlson comorbidity index; median (IQR)	2 (1-3)	2 (1-3)	2 (1-3)	0.841^†^
Preoperative eGFR (mL/min/1.73m^2^); mean ± standard deviation	96.1 (27)	95.3 (29.4)	96.1 (26.8)	0.909^‡^
Preoperative creatinine, (mg/dL); median (IQR)	0.8 (0.67-1.01)	0.89 (0.75-1.07)	0.80 (0.67-1.01)	0.306^†^
Preoperative chronic kidney disease; No. (%)	15 (10.1)	1 (6.3)	14 (10.5)	0.591^§^
Preoperative anemia; No. (%)	29 (19.5)	4 (25)	25 (18.8)	0.516^§^
Preoperative diabetes mellitus; No. (%)	27 (18.1)	3 (18.8)	24 (18)	0.945^§^
Preoperative hypertension; No. (%)	59 (39.6)	6 (37.5)	53 (39.8)	0.856^§^
Female sex; No. (%)	55 (36.9)	3 (18.7)	52 (39.1)	0.111^§^
Symptomatic presentation; No. (%)	47 (31.5)	7 (43.7)	40 (30.1)	0.266^§^
Tumor size (cm); median (IQR)	3.58 (2.49-4.81)	4.91 (3.91-5.89)	3.29 (2.42-4.12)	<0.001^†^
Left tumor side; No. (%)	74 (49.7)	9 (56.3)	65 (48.9)	0.577^§^
Clinical tumor stage; No. (%)				
T1a	92 (61.7)	3 (18.8)	89 (66.9)	0.001^§^
T1b	46 (30.9)	10 62.4)	36 (27.1)	
T2	11 (7.4)	3 (18.8)	8 (6)	
R.E.N.A.L. score; No. (%)				
low	82 (55)	2 (12.4)	80 (60.2)	<0.001^§^
intermediate	55 (36.9)	7 (43.8)	48 (36.1)	
high	12 (8.1)	7 (43.8)	5 (3.7)	
PADUA score; No. (%)				
low	55 (36.9)	0 (0)	55 (41.4)	<0.001^§^
intermediate	55 (36.9)	4 (25)	51 (38.3)	
high	39 (26.2)	12 (75)	27 (20.3)	
SPARE score; No. (%)				
low	95 (63.8)	2 (12.5)	93 (69.9)	<0.001^§^
intermediate	49 (32.8)	11 (68.7)	38 (28.6)	
high	5 (3.4)	3 (18.8)	2 (1.5)	
DAP score; No. (%)				
low	59 (39.6)	1 (6.3)	58 (43.6)	0.004^§^
high	90 (60.4)	15 (93.7)	75 (56.4)	

*Abbreviations: IQR – Interquartile range; BMI – body mass index; eGFR – estimated glomerular filtration rate.

†Mann-Whitney U test.

‡t-test.

§χ^2^ test.

**Figure 2:**
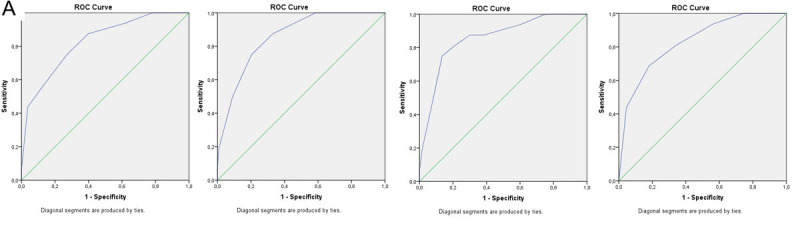
Receiver operating characteristic (ROC) curves for the power of nephrometry scores to predict the conversion to radical nephrectomy (RN). (**A**) R.E.N.A.L. score; (**B**) PADUA score; (**C**) SPARE score; (**D**) DAP score.

High R.E.N.A.L., PADUA, SPARE, and DAP scores were associated with the conversion to RN. In ROC analysis, the SPARE score had the highest area under curve (AUC = 0.807) (Figure 2, Table 2).

**Table 2:** Sensitivity, specificity, and threshold values of nephrometry scores in receiver operating characteristic (ROC) curve analysis

	Threshold	Sensitivity (%)	Specificity (%)	Area under the curve (95% confidence interval)	p
R.E.N.A.L.	7.5	75	72.9	0.740 (0.609-0.870)	0.002
PADUA	9.5	75	79.7	0.773 (0.644-0.903)	<0.001
SPARE	5.5	75	86.5	0.807 (0.679-0.936)	<0.001
DAP	7.5	68.8	82	0.754 (0.615-0.892)	0.001

In the univariate analysis, tumor size, clinical tumor stage, the R.E.N.A.L., PADUA, SPARE, and DAP score were associated with the conversion to RN (*P* < 0.001, *P* = 0.001, *P* < 0.001, *P* < 0.001, *P* < 0.001, *P* = 0.004, respectively). When evaluated individually in separate models in the multivariate analysis, the R.E.N.A.L, PADUA, SPARE and DAP scores were significantly associated with RN conversion (*P* = 0.009, *P* = 0.001, *P* < 0.001, *P* = 0.003, respectively). The SPARE score had the highest odds ratio (OR 12.561; confidence interval 3.456-45.534) (Table 3).

**Table 3:** Multivariate analysis of factors affecting the conversion to radical nephrectomy with models including each nephrometry score seperately

	Multivariate analysis	
	Odds ratio (95% confidence interval)	p
Model 1		
Tumor size	-	0.912
Clinical tumor stage T1b	5.206 (1.280-21.176)	0.035
Clinical tumor stage T2	8.178 (1.300-51.462)	
R.E.N.A.L. score >7.5	5.346 (1.530-18.676)	0.009
Model 2		
Tumor size	-	0.656
Clinical tumor stage T1b	4.629 (1.113-19.248)	0.047
Clinical tumor stage T2	8.300 (1.253-54.991)	
PADUA score >9.5	8.082 (2.272-28.756)	0.001
Model 3		
Tumor size	-	0.466
Clinical tumor stage T1b	-	0.110
Clinical tumor stage T2	-	
SPARE score >5.5	12.561 (3.456-45.534)	<0.001
Model 4		
Tumor size	-	0.897
Clinical tumor stage T1b	4.328 (1.026-18.262)	0.041
Clinical tumor stage T2	9.339 (1.446-60.317)	
DAP score >7.5	6.780 (1.958-23.485)	0.003

The number of patients with Clavien-Dindo grade 1, grade 2, grade 3a, and grade 4a complications was 4 (2.7%), 7 (4.7%), 3 (2%), and 2 (1.3%), respectively. Six patients (4%) required intraoperative and 6 patients (4%) required postoperative blood transfusion. Perioperative outcomes are shown in Table 4.

**Table 4:** Perioperative, pathological, functional, and oncological outcomes

**Parameters**	Conversion	No conversion
Operation time (min); median (IQR)	120 (102-142)	124 (101-150)
Hospitalization time (days); median (IQR)	3 (2.25-4)	3 (2-3)
Drain removal time (days); median (IQR)	2 (2-3)	2 (1-2)
Estimated blood loss (mL); median (IQR)	100 (35-375)	35 (0-100)
Intraoperative transfusion; No. (%)	3 (18.8)	3 (2.3)
Postoperative transfusion; No. (%)	3 (18.8)	3 (2.3)
Minor complications; No. (%)	3 (18.8)	8 (6)
Major complications; No. (%)	1 (6.3)	4 (3)
Malign pathology; No. (%)	15 (93.8)	99 (74.4)
Primary tumor stage; No. (%)		
T1	11 (68.7)	118 (88.7)
T2	0 (0)	3 (2.3)
T3	5 (31.3)	12 (9)
Nuclear grade; No. (%)^†^		
low	9 (69.2)	80 (86)
high	4 (30.8)	13 (14)
Lymphovascular invasion; No. (%)	3 (18.8)	7 (5.3)
Latest eGFR (mL/min/1.73m^2^); mean (±standard deviation)	66.4 (18.1)	89.6 (33.6)
Latest creatinine (mg/dL); median (IQR)	1.2 (0.95-1.47)	0.86 (0.72-1.12)

*IQR – interquartile range.

†Nuclear grade was reported for 93 and 13 patients who underwent partial nephrectomy and conversion to radical nephrectomy, respectively.

Histopathological examination revealed malignant pathology in 114 (76.5%) patients, the majority of whom had clear cell RCC (n = 72, 48.3%). Pathological, oncological, and functional outcomes are shown in Table 4.

In the follow-up period, 2 patients had local recurrence, while 2 patients developed distant metastases. Local recurrences were observed 15 and 24 months after PN. Among patients with local recurrences, one patient underwent surgical treatment and another patient was prescribed tyrosine kinase inhibitors after surgical treatment of recurrence. Tyrosine kinase inhibitors were initiated in patients with metastatic disease.

## DISCUSSION

In the present study, a high SPARE score was significantly associated with the conversion to RN in patients who underwent open PN.

The rates of conversion to RN vary widely according to surgeon's experience, center's experience, and the procedure type (17-21). The conversion to RN rate in this open PN study was slightly higher than those reported in other open PN series (18,19). Over the years, the PN application has expanded to patients with more complex renal masses. The literature reports that PN can be applied in patients with T1b and T2 tumors (3,22,23). T1b and T2 tumor rates in the present study were higher compared with those in a multi-institutional study by Arora et al (20). This difference might have affected the high conversion rate observed in the current study. Our hospital being a urooncology referral center may explain the observed high tumor size, T1b and T2 tumors rates, and the conversion to RN rate.

The conversion to RN can be caused by various factors, especially oncological concerns. In the present study, the main reasons for the conversion were hilar invasion, suspected advanced disease, and tumor size discordance. Positive surgical margins after PN are reported to be significantly associated with worse overall survival (24). In agreement with oncological principles, pT3 rate was significantly higher in the converted group.

Among the nephrometry scores analyzed in the present study, the SPARE had the highest AUC in ROC analysis and the highest OR for the conversion to RN. The SPARE score was developed by Ficarra et al by simplifying the PADUA score (12) with an aim to predict postoperative complications. We, on the other hand, used the SPARE score to predict the conversion to RN, which is an intraoperative complication. The main advantage of the SPARE score over other nephrometry scores for the prediction of the conversion to RN is the inclusion of more factors that can separately lead to the conversion to RN. Therefore, we believe that the SPARE score might be useful in the preoperative evaluation of the conversion to RN.

In line with previously published studies (17-19), in our study tumor size was a significant predictor of the conversion to RN in the univariate analysis, but in the multivariate analysis this effect disappeared. Notably, the rate of the conversion to RN is expected to increase with the increasing tumor size.

Unlike in other studies, preoperative kidney function in our study was not predictive for the conversion to RN (17,18). This difference may be explained by performing PN to protect the existing kidney function and by tumors not suitable for PN in patients with poor basal kidney function. In our study, 14 patients with preoperative CKD underwent successful PN, and of these patients, 3 had cT2a disease. Therefore, PN should be performed in all patients with a tumor suitable for PN regardless of preoperative kidney function.

The study limitations include the retrospective design, relatively small sample size, and single-center setting. Since all surgeons in our study had a high level of experience in urooncology (≥200 procedures), surgical experience was not included as a parameter. Another limitation is that nephrometry scores were calculated only by a single urologist. However, it was shown that physicians without specialized radiological training are equally successful in the calculation of nephrometry scores as board certified radiologists (25,26). The inclusion of patients who were operated on with open surgical technique can be considered as a limitation as well. However, European Association of Urology guidelines state that PN can be performed with open, laparoscopic, or robotic approaches, with the treatment choice being based on surgeon’s expertise and skills (27). Besides, laparoscopic and robotic PN have been shown to have a longer learning curve than open PN (28). Given our center's great experience in performing open PN and the fact that all surgeons in our center did not complete the learning curve for minimally invasive procedures, only patients who underwent open surgery were evaluated. Despite these limitations, we believe that the results of our study add to the current literature, as to the best of our knowledge, this is the first study comparing four different nephrometry scores and showing that SPARE score can also be used in predicting the conversion to RN.

In conclusion, we showed that a high SPARE score (>5.5) was an independent prognostic factor for predicting the conversion to RN in patients undergoing open PN. This nephrometry score may be used to inform the patients preoperatively about the risk of the conversion to RN during the surgical procedure. Additional studies are needed to corroborate our findings.
